# Metabolomics revealed mechanism for the synergistic effect of sulbactam, polymyxin-B and amikacin combination against *Acinetobacter baumannii*

**DOI:** 10.3389/fmicb.2023.1217270

**Published:** 2023-06-29

**Authors:** Shixing Zhu, Jiali Yue, Xintong Wang, Jiayuan Zhang, Mingming Yu, Yuanchao Zhan, Yuanqi Zhu, Sherwin K. B. Sy, Zhihua Lv

**Affiliations:** ^1^Ocean University of China, Qingdao, China; ^2^Laboratory for Marine Drugs and Bioproducts of Qingdao National Laboratory for Marine Science and Technology, Qingdao, China; ^3^Department of Laboratory Medicine, The Affiliated Hospital of Qingdao University, Qingdao, China; ^4^Department of Statistics, State University of Maringá, Maringá, Paraná, Brazil

**Keywords:** *Acinetobacter baumannii*, metabolomics, polymyxin-B, amikacin, sulbactam significance

## Abstract

**Introduction:**

The emergence of multidrug-resistant (MDR) *Acinetobacter baumannii* prompts clinicians to consider treating these infections with polymyxin combination.

**Methods:**

Metabolomic analysis was applied to investigate the synergistic effects of polymyxin-B, amikacin and sulbactam combination therapy against MDR *A. baumannii* harboring OXA-23 and other drug resistant genes. The drug concentrations tested were based on their clinical breakpoints: polymyxin-B (2 mg/L), amikacin (16 mg/L), polymyxin-B/amikacin (2/16 mg/L), and polymyxin-B/amikacin/sulbactam (2/16/4 mg/L).

**Results:**

The triple antibiotic combination significantly disrupted levels of metabolites involved in cell outer membrane structure including fatty acids, glycerophospholipids, nucleotides, amino acids and peptides as early as 15 min after administration. Amikacin and polymyxin-B alone perturbed a large number of metabolites at 15 min and 1 h, respectively, but the changes in metabolites were short-lived lasting for less than 4 h. In contrast, the combination treatment disrupted a large amount of metabolites beyond 4 h. Compared to the double-combination, the addition of sulbactam to polymyxin-B/amikacin combination produce a greater disorder in *A. baumannii* metabolome that further confer susceptibility of bacteria to the antibiotics.

**Conclusion:**

The metabolomic analysis identified mechanisms responsible for the synergistic activities of polymyxin-B/amikacin/sulbactam against MDR *A. baumannii*.

## Introduction

*Acinetobacter baumannii* is a tenacious gram-negative nosocomial pathogen that can survive a wide range of temperatures, pH, and humidity (Gootz and Marra, 2008) and often causes hospital-acquired and ventilator-associated pneumonia (HAP/VAP), urinary tract infection, blood flow infection, meningitis, skin and soft tissue infections in critically ill patients (Clark et al., 2016; Freire et al., 2016). Over the past few decades, the number of community-acquired infections caused by *A. baumannii* has been steadily increasing, resulting in high morbidity and mortality (Dijkshoorn et al., 2007; Sengstock et al., 2010). Multidrug-resistant (MDR) strains have spread from areas with high antibiotic resistance to areas with low rates of antibiotic resistance (Perez et al., 2007).

Many broad-spectrum antibiotics are ineffective against MDR *A. baumannii* (Avedissian et al., 2019). Since early 2000s, polymyxins have been used to treat many infections caused by MDR *A. baumannii* (Li et al., 2006; Poirel et al., 2017). *A. baumannii* confers resistance to polymyxin-B through modification of lipid A or single nucleotide mutations leading to a complete loss of lipopolysaccharide (LPS) (Girardello et al., 2017). Resistance to colistin alone has been reported (Zhao et al., 2022). Monotherapy can no longer meet the clinical needs, and the combination of polymyxin-B and other antibiotics is needed to improve the survival rate of patients infected with MDR *A. baumannii* (Oo and Sy, 2018, 2020; Wang et al., 2023). There have been reports of improved results of polymyxin combination therapy in the clinic (Teo et al., 2015; Sharma et al., 2017; Hussein et al., 2019a). Even though sulbactam is primarily used as a β-lactamase inhibitor, it has inherent antibacterial activity against *A. baumannii* and is one of the main drugs to treat *Acinetobacter* infections, often in combination with other antibiotics (Penwell et al., 2015; Deng et al., 2022). In the absence of a partnering β-lactam, sulbactam can potentially enhance the activity of polymyxin-B by making the cell wall leaky.

We have previously shown that even though polymyxin-B/amikacin/sulbactam combination can exert synergistic activities against *A. baumannii*, patients can benefit from localized administration in addition to intravenous route to ensure sufficient drug concentrations at the site of infection such as the lung (Zhu et al., 2022a,b; Zhang et al., 2023). Due to the poor penetration of both amikacin and polymyxin-B into the lung, inhaled administration of these two drugs can improve probability of treatment success in HAP/VAP. As an extension of our previous work, we performed metabolomic analysis to understand mechanisms responsible for the synergistic activities of polymyxin-B in combination with amikacin and sulbactam against *A. baumannii*.

## Materials and methods

### Antibiotics, reagents and bacterial isolates

Polymyxin-B sulfate, amikacin, and sulbactam (Shanghai Macklin Biochemical Co., Ltd., Shanghai, China) solutions were prepared according to the Clinical and Laboratory Standards Institute (CLSI) guidelines (CLSI, 2020). The stock solutions of polymyxin-B sulfate, amikacin, and sulbactam were dissolved in pure water at a concentration of 5210 μg/mL for all three antibiotics and stored at −80°C. Working solutions were diluted in Millipore water and filtered before use. Clinical *A. baumannii* isolate 12 was obtained from the affiliated hospital of Qingdao University and grown in cation-adjusted Mueller-Hinton broth (CAMHB; LAND BRIDGE, Beijing, China). The quality control strain for antimicrobial susceptibility test was *E. coli* ATCC 25922 and *A. baumannii* ATCC 19606. The resistance genes carried by the clinical isolate 12 are shown in Table 1.

**Table 1:** Minimum inhibitory concentrations (MIC) of amikacin, polymyxin-B and sulbactam alone or in combination with or without sulbactam (4 mg/L) agains *A. baumannii* isolate 12, as well as drug resistance genes encoded.

Strains	Drug resistance genes encoded	MIC (mg/L)						
		Amikacin	Polymyxin-B	Sulbactam	Amikacin/sulbactam	Polymyxin-B/sulbactam	Amikacin/polymyxin-B	Amikacin/polymyxin-B/sulbactam
Control								
*E. coli ATCC19606*		1	1	32	–	–	–	–
*A. baumannii*								
12	*bla*OXA23; *bla*ADC25; *bla*TEM-1D; *bla*OXA66; *sul1; sul2; tet(B); aph(3″)-b; aph(3″)-lb;aph(3′)-la;aph(6)-ld; aph(3″)b*	>128	16	>64	>128/4	8/4	4/2	2/2/4

MIC, minimum inhibitory concentration; CLSI and EUCAST breakpoints for interpretation of amikacin MIC results: ≤4 mg/L (susceptible), and < 4 mg/L (resistant); and polymyxin-B MIC results: ≤2 mg/L (intermediate), >2 mg/L (resistant) for *A. baumannii*.

### *In vitro* susceptibility testing

The broth microdilution method was used to determine the MIC of various antibiotics against *A. baumannii* isolate 12, based on the CLSI guidelines (CLSI, 2020). Antibiotics were added in two-fold increment in each well of the sterile 96-well plate. The *A. baumannii* isolate 12 was cultured to a density of 0.5 McFarland and then diluted into each well of the 96-well plate to a final concentration of approximately 5 × 10^5^ CFU/mL; the plate was then placed in a constant temperature incubator at 35 ± 2°C for 20 h. All MIC determination were performed in triplicate.

### Bacteria culture preparation for metabolomic profiling

A single colony of *A. baumannii* isolate 12 was inoculated into 15 mL fresh MHB and incubated at 37°C overnight and then diluted 100-folds into 200 mL fresh MHB and shaking at 180 rpm (37°C). After the bacteria were cultured to logarithmic growth stage with an optical density (OD_600_) of approximately 0.5, antibiotics were added (Maifiah et al., 2017). The culture was divided into five treatment groups: (1) control without any antibiotics (AB-control); (2) polymyxin-B alone (2 μg/mL); (3) amikacin alone (16 μg/mL); (4) the combination of polymyxin-B and amikacin (2/16 μg/mL); and (5) the combination of polymyxin-B, amikacin, and sulbactam (2/16/4 μg/mL). Our previous study had shown that the triple combination could reduce MIC to or below the clinical breakpoints (Zhu et al., 2022a). The antibiotic concentrations were chosen based on CLSI and EUCAST breakpoints, which are associated with realistic pharmacokinetic concentrations in the clinic. In 2023, the CLSI lowered the Enterobacterales susceptibility breakpoint for amikacin from ≤16 mg/L to ≤4 mg/L (Sader et al., 2023). There is no susceptible breakpoint for polymyxin-B and the intermediate breakpoint was used (Satlin et al., 2020). Five replicates were prepared for each treatment. Samples were taken at three timepoints of 15 min, 1 h and 4 h and normalized to an OD_600_ of 0.5 before extraction (Zhu et al., 2022c).

### Preparation of cellular metabolite extracts

Cellular metabolites of *A. baumannii* were extracted by previously reported methods (Hussein et al., 2019b; Zhao et al., 2021). Firstly, samples were centrifuged at 3,220 × *g* at 4°C for 10 min, then the bacterial pellets were washed with cold saline twice. 500 μL cold chloroform-methanol–water (CMW) (1:3:1 by volume) solution containing 1 μM of each of the internal standards (CHAPS, CAPS, PIPES, and Tris) was added. The samples were flash frozen in liquid nitrogen and thawed on ice, and then fully vortexed. The samples were then centrifuged for 10 min at 3,220 × *g* at 4°C to remove cell debris, and then 300 μL of the supernatants were added to 1.5 mL Eppendorf tubes for centrifugation. After centrifugation at 14,000 × *g* at 4°C for 10 min, 200 μL of supernatant was transferred into injection vials for analysis by liquid chromatography-high resolution mass spectrometry (LC–MS). Quality control (QC) samples were obtained by pooling the samples, and their cellular metabolites were extracted as described above.

### LC-MS analysis

The LC–MS method was established based on a previously reported method with modifications (Zhu et al., 2022c). Samples were analyzed by LC–MS equipped with an Ultimate 3,000 ultra-high-performance liquid chromatography (UHPLC) system (Thermo Scientific, CA, United States) and Q-Exactive Orbitrap mass spectrometer (Thermo Scientific, CA, United States) operated in both positive and negative electrospray ionization (ESI) modes (polarity switching) at 35,000 resolution with a detection range of *m/z* 50 to 1,275 Da. The separation was performed on a HILIC column (2.1 × 100 mm, 1.7 μm; ACE 1.7 μm, HILIC-A, United Kingdom) coupled with guard column (ACE UHPLC pre-column filter) operated at 40°C. The mobile phase which consisted of 10 mM ammonium acetate in water (solvent A), and acetonitrile (solvent B) was delivered at a flow rate of 0.3 mL/min. The gradient elution mode started from 80 to 20% over 15 min, then to 5% of solvent B at 18 min, followed by a wash with 5% B for 3 min, and re-equilibration for 8 min with 80% B. The injection volume was 10 μL.

### Data processing, bioinformatics, and statistical analyses

The raw data file obtained by LC–MS analysis was analyzed by Progenesis QI program (Waters, Mass, United States). The workflow was established for importing data, automatic alignment, peak selection based on the minimum intensity of 50,000, and subsequent identification of metabolites. Metabolites were identified by LC retention time and accurate *m/z* value. The maximum retention time shift for peak alignment was 0.2 min, and the mass tolerance was 5 ppm. Metabolites were normalized by the mean, log_10_-transformed and auto scaling. Statistical analysis was performed using MetaboAnalyst 5.0 web portal.^1^ Principal component analysis (PCA) was performed for all groups at each time point. Student’s *t*-test was used to determine significantly altered metabolites (*p* < 0.05). False discovery rate (FDR) <0.05 and fold change (FC) ≥2 (log_2_FC ≥ 1 or ≤ −1) were also evaluated by the algorithm. Metabolites and pathway analysis were searched using KEGG (Kyoto Encyclopedia of Genes and Genomes) and HMDB (The Human Metabolome Database) databases.

## Results

### *In vitro* susceptibility evaluation of *Acinetobacter baumannii* isolate 12 to common antibiotics and polymyxin-B/amikacin/sulbactam combination

Table 2 lists the *in vitro* susceptibility results of *A. baumannii* isolate 12 to commonly used antibiotics. This isolate is resistant to common antibiotics used in the clinic, including amikacin and polymyxin-B (Table 1). The addition of 4 μg/mL sulbactam did not reduce the MIC of amikacin monotherapy, whereas the polymyxin-B MIC was reduced from 16 to 8 μg/mL in the presence of 4 μg/mL sulbactam. The MIC values of each drug in amikacin/polymyxin-B and amikacin/polymyxin-B/sulbactam combination had significant reduction from their monotherapy MICs.

**Table 2:** Minimum inhibitory concentrations (MIC) of commonly used clinical antibiotics against *Acinetobacter baumannii* isolate 12.

Antibiotics	MIC (mg/L)	Antibiotics	MIC (mg/L)
Meropenem	16	Sulfamethoxazole	>64
Levofloxacin	>16	Vancomycin	>128
Minocyline	32	Ciprofloxacin	>64
Cefepime	>32	Clindamycin	>64
Rifampin	8	Ampicillin	>64
Fosfomycin	128	Cefoperazone	>64
Gentamicin	32	Ceftriaxone	>8
Azithromycin	16	Doxycycline	8
Daptomycin	>64	Clarithromycin	4
Ampicillin	>128	Linezolid	>16
Fluconazole	>64	Metronidazole	>64
Piperacillin-Tazobactam	>64	Moxifloxacin	32
Cefoxitin	>64	Clavulanate	>64
Ceftazidime	>64	Imipenem	>64
Aztreonam	64		

### The metabolic spectra against *Acinetobacter baumannii* isolate 12

**Figure 1:**
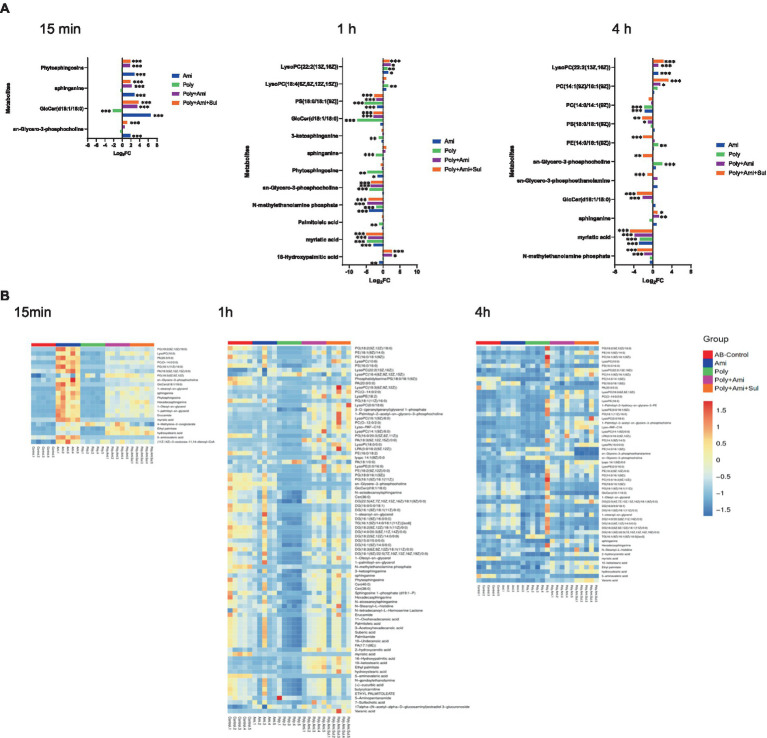
Principal component analysis **(A)** and heatmap **(B)** profiles of MDR *A. baumannii* treated with amikacin and polymyxin-B as monotherapy and in combination with and without sulbactam at 15 min, 1 h and 4 h.

In the present study, a total of 555 metabolites in bacteria extract were identified by KEGG and HMDB databases. Among them, 73 were associated with known metabolic pathways. PCA was performed to delineate putative metabolites that were affected by differential effects of drugs on the *A. baumannii* isolate (Figure 1). After 15 min of treatment, polymyxin-B, amikacin, and the combination groups were clustered and were significantly distanced from the control group. However, there was an overlap between polymyxin-B/amikacin and polymyxin-B/amikacin/sulbactam combination groups. Even though there were differences characterized by the distance between the monotherapies and control group after 1 h and 4 h treatment, their distances started to shrink, whereas the clusters representing the two combination therapies were quite distinct from the control group. At 4 h treatment, there was a clear separation between polymyxin-B/amikacin and polymyxin-B/amikacin/sulbactam treatment groups.

All altered metabolites were grouped by metabolic pathways, which were assigned to lipids, amino sugars, nucleotides, amino acids and central carbon metabolism. The affected metabolic pathways were primarily involved in the metabolisms of lipids, nucleotides and amino acids.

### Lipid metabolism pathway was significantly affected by polymyxin-B plus amikacin with or without sulbactam in *Acinetobacter baumannii*

**Figure 2:**
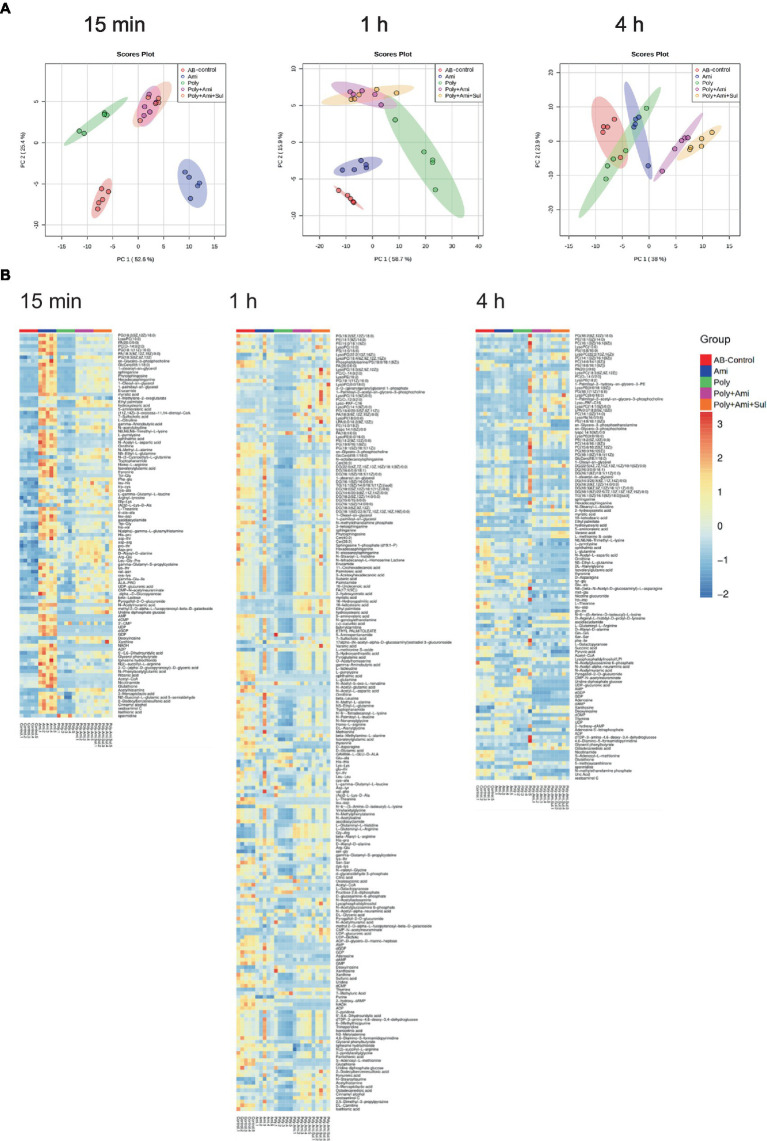
Perturbations of lipid metabolites in *A. baumannii* treated with antibiotics alone and in combination at 15 min, 1 h and 4 h. **(A)** Bar charts show the log_2_FC values of metabolites associated with lipid pathway. **(B)** Heatmap shows the normalized relative intensity of the significantly disrupted metabolites involved in lipid metabolism (*p* value < 0.05, log_2_FC ≤ −1 or log_2_FC ≥ 1, FDR < 0.05). *p < 0.05; **p < 0.01; ***p < 0.001.

A number of lipid molecules were affected by the combination therapy, as illustrated in Figures 2A,B. Among them, sn-glycero-3-phosphocholine, sn-glycero-3-phosphoethanolamine and N-methylethanolamine phosphate were three important intermediates for outer membrane structure. The level of sn-glycero-3-phosphocholine (log_2_FC = −3.5 to1.1) was remarkably affected by polymyxin-B/amikacin/sulbactam at the three time points. The level of N-methylethanolamine phosphate was significantly depleted at 1 h and 4 h (log_2_FC = −4.2 to −3.2). However, the level of sn-glycero-3-phosphoethanolamine (log_2_FC = −1.1) was reduced only at 4 h.

In addition, the polymyxin-B/amikacin/sulbactam combination perturbed the levels of sphinganine and glucosylceramide (d18:1/18:0; log_2_FC = −3.3 to 3.8), which were two important intermediates associated with the sphingolipid pathway, at 15 min and 4 h, respectively. Perturbation of metabolites associated with the fatty acid pathway occurred after 1 h in all four treatment conditions. Myristic acid and 16-hydroxypalmitic acid were two intermediates in the fatty acid pathway that were affected. The level of myristic acid (log_2_FC = −4.9 to −4.8) was reduced by polymyxin-B/amikacin/sulbactam combination at 1 h and 4 h. In addition, the level of 16-hydroxypalmitic acid (log_2_FC = 2.6) was also increased at 1 h. In contrast, the level of palmitoleic acid (log_2_FC = −1.2) was only depleted by polymyxin-B monotherapy at 1 h.

The results showed that amikacin and polymyxin-B alone caused lipid metabolism disorder at an early stage, but the effect was weakened after 4 h. While the disturbance induced by polymyxin-B/amikacin treatment was similar to that of polymyxin-B/amikacin/sulbactam treatment at 1 h, the effect was weaker in the polymyxin-B/amikacin group than that in the polymyxin-B/amikacin/sulbactam combination at 4 h (Figures 2A,B).

### Polymyxin-B plus amikacin with or without sulbactam disrupted amino sugar, lipopolysaccharide (LPS), peptidoglycan and central carbon metabolism (CCM) pathways

**Figure 3:**
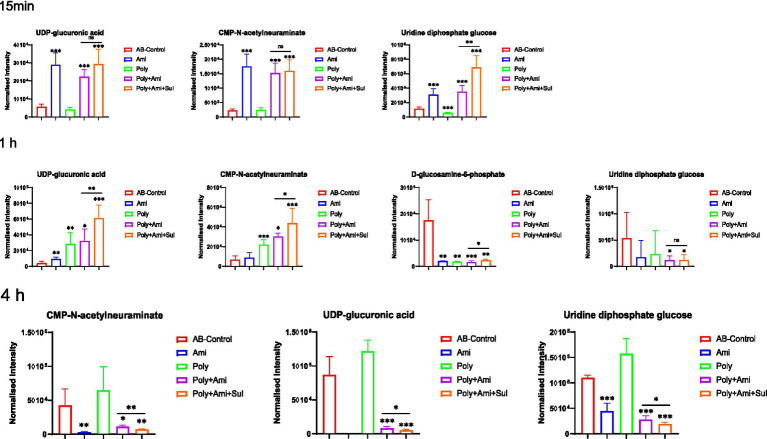
Perturbations of metabolites associated with LPS, peptidoglycan and amino sugar pathway in *A. baumannii* treated with antibiotics alone and the combination at 15 min, 1 h and 4 h. Box plots show the differences of normalized relative intensity of the metabolites involved in pathways (*p* value < 0.05, log_2_FC ≤ −1 or log_2_FC ≥ 1, FDR < 0.05). *p < 0.05; **p < 0.01; ***p < 0.001.

**Figure 4:**
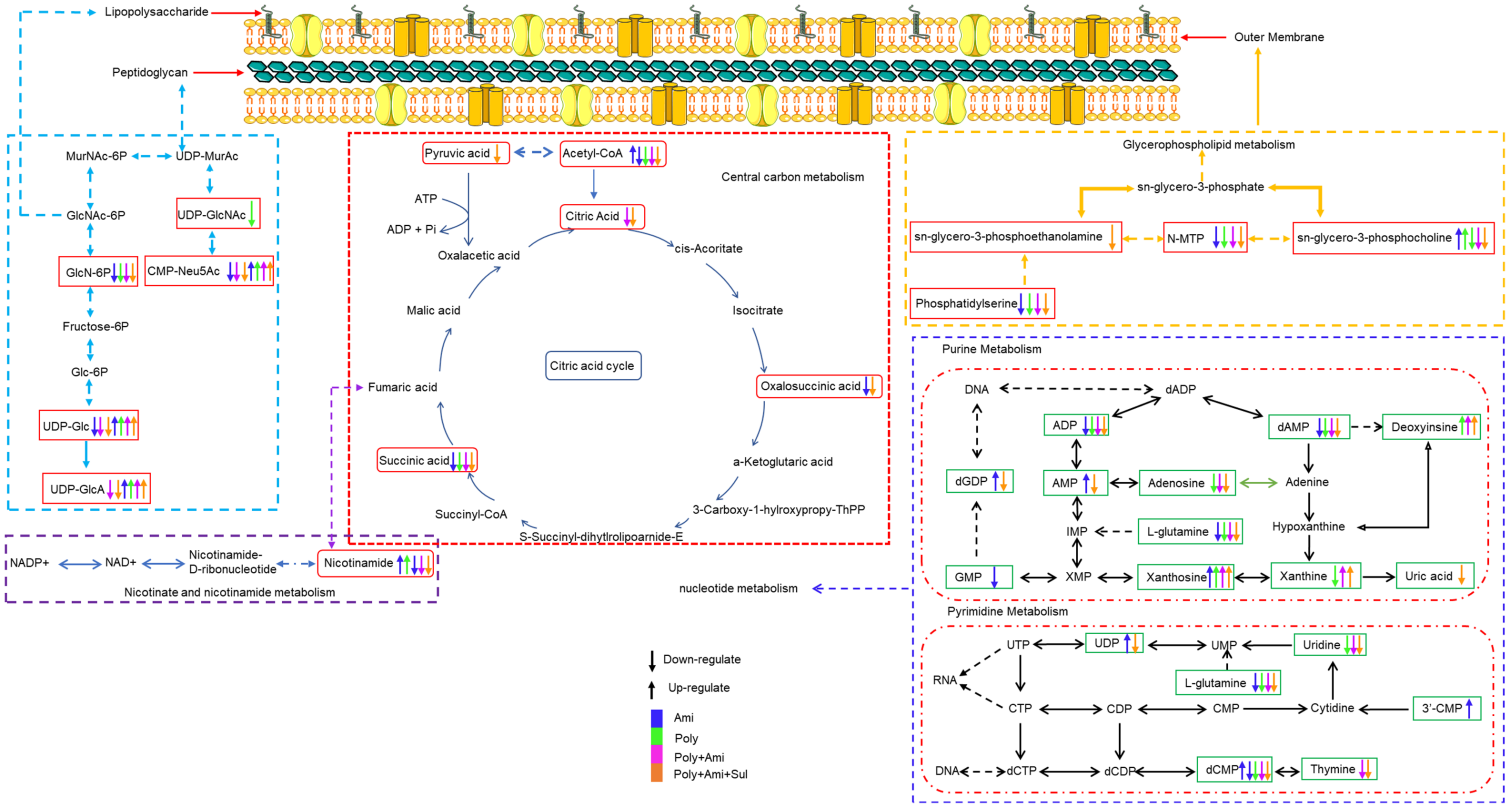
Illustration of affected metabolomic pathways in *A. baumannii* treated with antibiotics as monotherapy and combination therapy.

The polymyxin-B/amikacin/sulbactam combination significantly perturbed the levels of UDP-glucuronic acid (UDP-GlcA), CMP-N-acetylneuraminate (CMP-Neu5Ac), and uridine diphosphate glucose (UDP-Glc) (log_2_FC = −4.0 to 3.9), which are the three essential intermediates involved in amino sugars, lipopolysaccharides and peptidoglycans pathways (Figures 3, 4). The levels of D-glucosamine-6-phosphate (GlcN-6P, log_2_FC = −2.9) were reduced at 1 h.

The disturbances induced by polymyxin-B/amikacin combination were similar but weakened compared to the polymyxin-B/amikacin/sulbactam combination (Figure 3). For monotherapy, amikacin-induced metabolite disorder was generally weaker than that in the combination treatments at 1 h after administration. Polymyxin-B alone caused metabolic changes primarily at 1 h (Figure 3).

**Figure 5:**
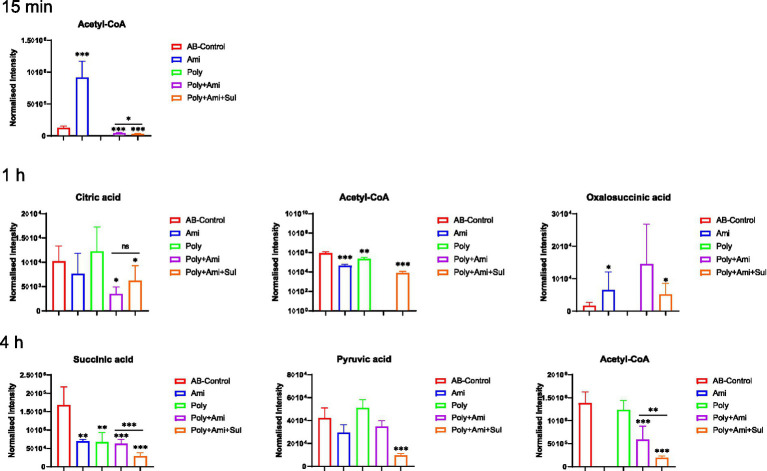
Perturbations of central carbon metabolism in *A. baumannii* treated with antibiotics alone and the combination at 15 min, 1 h and 4 h. Box plots show the differences of normalized relative intensity of the metabolites involved in CCM pathways (*p*-value < 0.05, log_2_FC ≤ −1 or log_2_FC ≥ 1, FDR < 0.05). *p < 0.05; **p < 0.01; ***p < 0.001.

The central carbon metabolism (CCM) pathway is slightly affected at the three timepoints but combination therapies showed greater differences compared with the monotherapies (Figure 5). The triple-combination treatment significantly reduced the level of acetyl-CoA (log_2_FC = −6.8 to −2.1), which is an essential intermediate for CCM, fatty acid and glutathione pathways. In addition, polymyxin-B/amikacin/sulbactam combination therapy disturbed the levels of citric acid/oxalosuccinic acid (log_2_FC = 1.2/1.6) and succinic acid/pyruvic acid (log_2_FC = −2.5/ −2.1) at 1 h and 4 h, respectively. The changes induced by the polymyxin-B/amikacin combination and monotherapy treatments were weaker than that of polymyxin-B/amikacin/sulbactam combination in the CCM pathway (Figure 5).

### Polymyxin-B plus amikacin with or without sulbactam treatments altered nucleotide, nicotinate, and nicotinamide metabolic pathway

The affected nucleotide metabolic pathway mainly included pyrimidine and purine. For the pyrimidine metabolism, three key affected metabolites by polymyxin-B/amikacin/sulbactam (log_2_FC = −4.3/−2.1/1.2 at 1 h and log_2_FC = −2.9/−1.2/2.3 at 4 h, respectively) and polymyxin-B/amikacin (log_2_FC = −5.6/−2.6/1.3 at 1 h) combinations were dCMP, uridine and thymine. The magnitude of metabolite changes due to polymyxin-B/amikacin was smaller compared to that of polymyxin-B/amikacin/sulbactam, especially at 15 min and 4 h.

For monotherapy, only amikacin perturbed the levels of dCMP, 3’-CMP and UDP (log_2_FC = 1.4 to 1.9) at 15 min. Polymyxin-B did not induce obvious disturbance of metabolites related to the pyrimidine metabolic pathway.

Both monotherapies and combination therapies affected the purine metabolic pathway, which primarily occurred after 1 h of treatment. The affected metabolites due to combination therapies were adenosine, AMP, GDP, dGDP, dAMP, ADP, deoxyinosine, uric acid, xanthosine; these changes were of varying degrees depending on time and the type of combination treatment.

**Figure 6:**
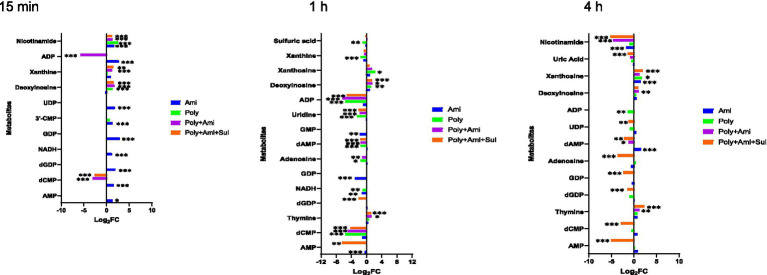
Perturbations of metabolites associated with nucleotide and derivative metabolism in *A. baumannii* treated with antibiotics alone and the combination at 15 min, 1 h and 4 h. Bar charts show the log_2_FC values of metabolites associated with pathway (*p* value < 0.05, log_2_FC ≤ −1 or log_2_FC ≥ 1, FDR < 0.05). *p < 0.05; **p < 0.01; ***p < 0.001.

For monotherapy, amikacin decreased the levels of NADH, GDP and GMP (log_2_FC = −1.3 to −3.1), whereas polymyxin-B significantly reduced the levels of 8 metabolites, including NADH, adenosine, dAMP, xanthine, ADP, sulfuric acid, deoxyinosine and xanthosine (log_2_FC = −5.7 to 2.2) at 1 h. At 4 h, the effect of monotherapy is significantly reduced (Figure 6).

The combination treatments also remarkably reduced the level of nicotinamide (log_2_FC = −5.2/−4.6), which is a key metabolite involved in nicotinate and nicotinamide metabolic pathway (Figures 4, 5).

### Amino acid and peptide metabolic pathways were significantly affected by polymyxin-B plus amikacin with or without sulbactam

**Figure 7:**
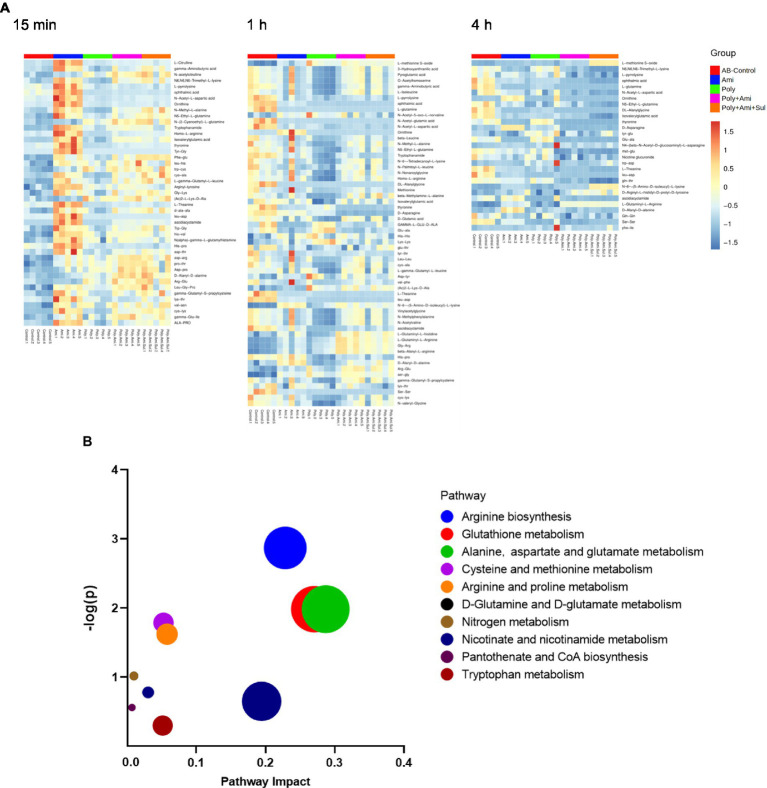
Heat map **(A)** profiles of normalized relative intensity significantly perturbed metabolites in amino acid and peptide in *A. baumannii* treated with antibiotics alone and the combination at 15 min, 1 h and 4 h (*p* value < 0.05, log_2_FC ≤ −1 or log_2_FC ≥ 1 and FDR < 0.05). Enrichment analysis **(B)** of amino acid metabolism pathway.

A large number of amino acids and peptides were affected by both monotherapies and combination therapies (Figure 7A). The metabolic pathways of the affected metabolites include arginine biosynthesis, glutathione, alanine, aspartate, and glutamate metabolism (Figure 7B).

The effects of the four treatments varied with time. Amikacin was driving the most changes in metabolites at 15 min. The levels of 10 key metabolites including L-citrulline, 5-aminovaleric acid, gamma-aminobutyric acid, N6,N6,N6-trimethyl-L-lysine, L-pyrrolysine, glutathione, ophthalmic acid, N-acetyl-L-aspartic acid, 3-mercaptolactic acid, and N2-succinyl-L-glutamic acid 5-semialdehyde (log_2_FC = 1.2 to 2.8) were significantly disturbed. At this time point, polymyxin-B/amikacin/sulbactam and polymyxin-B/amikacin treatments induced perturbations of 6 and 5 metabolites (log_2_FC = −2.3 to 2.5/ log_2_FC = −2.4 to 2.2), respectively.

At 1 h, polymyxin-B caused changes in a total of 18 metabolites including 5-aminovaleric acid, L-methionine S-oxide, 3-hydroxyanthranilic acid, pyroglutamic acid, O-acetylhomoserine, gamma-aminobutyric acid, L-isoleucine, L-pyrrolysine, ophthalmic acid, L-glutamine, N-acetyl-5-oxo-L-norvaline, N-acetyl-glutamic acid, N-acetyl-L-aspartic acid, pantothenic acid, S-adenosyl-L-methionine, glutathione, kynurenic acid and 3-mercaptolactic acid (log_2_FC = −6.8 to 1.2). For combination therapies, 10 and 11 key metabolites (log_2_FC = −5.8 to 1.1/log_2_FC = −6.3 to 1.0) were significantly affected by polymyxin-B/amikacin and polymyxin-B/amikacin/sulbactam, respectively (Table 3).

**Table 3:** Sequence of metabolomic changes in *A. baumannii* after polymyxin-B and amikacin treatments as monotherapy and in combination with and without sulbactam.

Time	Polymyxin-B/amikacin/sulbactam	Polymyxin-B/amikacin	Polymyxin-B	Amikacin
15 min	Cell wall synthesis ↑UDP-glucuronic acid; ↑CMP-N-acetylneuraminate; ↑uridine diphosphate glucose	Cell wall synthesis ↑UDP-glucuronic acid; ↑CMP-N-acetylneuraminate; ↑uridine diphosphate glucose	Cell wall synthesis ↑uridine diphosphate glucose	Cell wall synthesis ↑UDP-glucuronic acid; ↑CMP-N-acetylneuraminate
	Outer membrane glycerophospholipids ↓sn-glycero-3-phosphocholine	Outer membrane glycerophospholipids NA	Outer membrane glycerophospholipids NA	Outer membrane glycerophospholipids ↓sn-glycero-3-phosphocholine
	Central carbon metabolism pathway ↓acetyl-CoA	Central carbon metabolism pathway ↓acetyl-CoA	Central carbon metabolism pathway NA	Central carbon metabolism pathway ↑acetyl-CoA
	Nucleotide, nicotinate and nicotinamide pathway ↓dCMP; ↑deoxyinosine; ↑xanthine; ↑nicotinamide	Nucleotide, nicotinate and nicotinamide pathway ↑deoxyinosine; ↑xanthine; ↑dCMP; ↓ADP; ↑nicotinamide	Nucleotide, nicotinate and nicotinamide pathway ↑deoxyinosine; ↑nicotinamide	Nucleotide, nicotinate and nicotinamide pathway ↑ADP; ↑AMP; ↑dCMP; ↑dGDP; ↑GDP; ↑3’-CMP; ↑UDP; ↑AMP; ↑nicotinamide
	Amino acid and peptide pathway ↑N-acetylcitrulline; ↑acetylhistamine; ↑3-mercaptolactic acid; ↑N6,N6,N6-trimethyl-L-lysine; ↓ophthalmic acid; ↓5-aminovaleric acid;	Amino acid and peptide pathway ↑N-acetylcitrulline; ↑acetylhistamine; ↑3-mercaptolactic acid; ↓ophthalmic acid; ↓5-aminovaleric acid	Amino acid and peptide pathway ↑5-aminovaleric acid; ↑glutathione; ↑ophthalmic acid; ↑spermidine; ↑acetylhistamine	Amino acid and peptide pathway ↑5-aminovaleric acid; ↑L-citrulline; ↑gamma-aminobutyric acid; ↑L-pyrrolysine; ↑N-acetyl-L-aspartic acid; ↑N6,N6,N6-trimethyl-L-lysine; ↑ophthalmic acid; ↑glutathione; ↑3-Mercaptolactic acid; ↑N2-succinyl-L-glutamic acid 5-semialdehyde
1 h	Cell wall synthesis ↓uridine diphosphate glucose; ↓D-glucosamine-6-phosphate; ↑UDP-glucuronic acid; ↑CMP-N-acetylneuraminate	Cell wall synthesis ↓uridine diphosphate glucose; ↓D-glucosamine-6-phosphate; ↑UDP-glucuronic acid; ↑CMP-N-acetylneuraminate	Cell wall synthesis ↓D-glucosamine-6-phosphate; ↓ADP-D-glycero-D-manno-heptose; ↓UDP-GlcNac; ↓UDP-glucuronic acid; ↑CMP-N-acetylneuraminate	Cell wall synthesis ↓D-glucosamine-6-phosphate; ↑UDP-glucuronic acid
	Outer membrane glycerophospholipids ↓sn-glycero-3-phosphocholine; ↓N-methylethanolamine phosphate	Outer membrane glycerophospholipids ↓sn-glycero-3-phosphocholine; ↓N-methylethanolamine phosphate	Outer membrane glycerophospholipids ↓sn-glycero-3-phosphocholine; ↓N-methylethanolamine phosphate	Outer membrane glycerophospholipids ↓N-methylethanolamine phosphate
	Central carbon metabolism pathway ↓citric acid; ↑oxalosuccinic acid; ↓acetyl-CoA	Central carbon metabolism pathway ↓citric acid	Central carbon metabolism pathway ↓acetyl-CoA	Central carbon metabolism pathway ↑oxalosuccinic acid
	Nucleotide, nicotinate and nicotinamide pathway ↓AMP; ↓ADP; ↓urdine; ↓dAMP; ↓dGDP; ↓dCMP; ↑deoxyinosine; ↑thymine	Nucleotide, nicotinate and nicotinamide pathway ↓ADP;↓urdine; ↓dAMP; ↓dCMP; ↓adenosine; ↑deoxyinosine; ↑thymine	Nucleotide, nicotinate and nicotinamide pathway ↓sulfuric acid; ↓xanthine; ↓ADP; ↓urdine; ↓dAMP; ↓adenosine; ↓ NADH; ↓dCMP; ↑deoxyinosine; ↑xanthosine	Nucleotide, nicotinate and nicotinamide pathway ↓GMP; ↓GDP; ↓NADH
	Amino acid and peptide pathway ↓5-aminovaleric acid; ↓ophthalmic acid; ↓5-aminopentanamide; ↓L-pyrrolysine; ↓L-glutamine; ↓N-acetyl-glutamic acid; ↓N-acetyl-L-aspartic acid; ↓glutathione; ↓S-adenosyl-L-methionine; ↑acetylhistamine; ↑L-methionine S-oxide	Amino acid and peptide pathway ↓5-aminovaleric acid; ↓ophthalmic acid; ↓5-aminopentanamide; ↓L-pyrrolysine; ↓L-glutamine; ↓N-acetyl-glutamic acid; ↓N-acetyl-L-aspartic acid; ↓glutathione; ↓S-adenosyl-L-methionine; ↑L-methionine S-oxide	Amino acid and peptide pathway ↓5-aminovaleric acid; ↓pantothenic acid; ↓S-adenosyl-L-methionine; ↓glutathione; ↓kynurenic acid; ↓3-mercaptolactic acid ↓pyroglutamic acid; ↓L-methionine S-oxide; ↓L-Isoleucine; ↓3-hydroxyanthranilic acid; ↓L-pyrrolysine; ↓O-acetylhomoserine; ↓ophthalmic acid; ↓N-acetyl-glutamic acid; ↓gamma-aminobutyric acid; ↓L-glutamine; ↓N-acetyl-L-aspartic acid; ↑N-acetyl-5-oxo-L-norvaline	Amino acid and peptide pathway ↓5-aminopentanamide; ↓L-pyrrolysine; ↓ophthalmic acid; ↓N-acetyl-glutamic acid; ↓N-acetyl-L-aspartic acid; ↓L-glutamine
4 h	Cell wall synthesis ↓UDP-glucuronic acid; ↓CMP-N-acetylneuraminate; ↓uridine diphosphate glucose	Cell wall synthesis ↓UDP-glucuronic acid; ↓CMP-N-acetylneuraminate; ↓uridine diphosphate glucose	Cell wall synthesis NA	Cell wall synthesis ↓UDP-glucuronic acid; ↓uridine diphosphate glucose
	Outer membrane glycerophospholipids ↓sn-glycero-3-phosphocholine; ↓sn-glycero-3-phosphoethanolamine; ↓N-methylethanolamine phosphate	Outer membrane glycerophospholipids ↓N-methylethanolamine phosphate	Outer membrane glycerophospholipids ↑sn-glycero-3-phosphocholine	Outer membrane glycerophospholipids NA
	Central carbon metabolism pathway ↓succinic acid; ↓pyruvic acid; ↓acetyl-CoA	Central carbon metabolism pathway ↓pyruvic acid; ↓acetyl-CoA	Central carbon metabolism pathway ↓succinic acid	Central carbon metabolism pathway ↓succinic acid
	Nucleotide, nicotinate and nicotinamide pathway ↓uric acid; ↓dCMP; ↓UDP; ↓dAMP; ↓adensoine; ↓GDP; ↓dGDP; ↓dCMP; ↓AMP; ↓nicotinamide; ↑deoxyinosine; ↑xanthosine; ↑thymine	Nucleotide, nicotinate and nicotinamide pathway ↓nicotinamide; ↓dAMP; ↑thymine	Nucleotide, nicotinate and nicotinamide pathway ↓ADP; ↑xanthosine	Nucleotide, nicotinate and nicotinamide pathway ↓nicotinamide; ↑xanthosine; ↑dAMP
	Amino acid and peptide pathway ↓5-aminovaleric acid; ↓ophthalmic acid; ↓N6,N6,N6-trimethyl-L-lysine; ↓L-pyrrolysine; ↓L-glutamine; ↓S-adenosyl-L-methionine; ↓N-acetyl-L-aspartic acid; ↓glutathione; ↑L-methionine S-oxide	Amino acid and peptide pathway ↓5-aminovaleric acid; ↓ophthalmic acid; ↓N6,N6,N6-trimethyl-L-lysine; ↓L-glutamine; ↑L-methionine S-oxide; ↓N-acetyl-L-aspartic acid; ↓glutathione; ↓S-adenosyl-L-methionine	Amino acid and peptide pathway ↓5-aminovaleric acid; ↓ophthalmic acid; ↓L-glutamine; ↓S-adenosyl-L-methionine; ↓glutathione	Amino acid and peptide pathway ↓5-aminovaleric acid; ↓ophthalmic acid; ↓N6,N6,N6-trimethyl-L-lysine; ↓L-glutamine; ↓S-adenosyl-L-methionine; ↑spermidine

At 4 h, the combination of polymyxin-B/amikacin/sulbactam induced the most disturbance for a total of 9 metabolites including 5-aminovaleric acid, L-methionine-S-oxide, N6,N6,N6-trimethyl-L-lysine, L-pyrrolysine, ophthalmic acid, L-glutamine, N-acetyl-L-aspartic acid, S-adenosyl-L-methionine and glutathione (log_2_FC = −8.5 to 2.7). The treatment of polymyxin-B/amikacin perturbed 8 metabolites (Table 3). At this time point, the effects of amikacin and polymyxin-B alone, which induced disturbances in the levels of 6 and 5 key metabolites (log_2_FC = −5.2 to −1.2/log_2_FC = −2.5 to 1.4), respectively (Table 3) were weaker than that during 15 min and 1 h post-treatment.

## Discussion

This clinical isolate carried OXA-23, aph(3′)-la and other resistance genes, which made it resistant to both sulbactam and amikacin (Umezawa, 1979; Yang et al., 2019). The monotherapy treatment against this MDR isolate would have required a large plasma amikacin exposure. However, the risk of human toxicity increases when the steady-state trough amikacin concentration is greater than 10 mg/L (Dijkstra et al., 2014; Xu et al., 2022). In this isolate, the combination of polymyxin-B/amikacin reduced amikacin MIC value from >128 μg/mL in monotherapy to 2–4 μg/mL with and without sulbactam, respectively (Table 1). Combination therapy confers susceptibility in this MDR isolate to each of the antimicrobial agents in the combination.

In a previous study, we evaluated the time course of bacterial dynamics of several *A. baumannii* isolates in response to amikacin, polymyxin-B alone and in combination with sulbactam (4 mg/L fixed) using time-kill assays (Zhu et al., 2022a). The results showed that amikacin/polymyxin-B combined with or without sulbactam could inhibit bacterial growth; the bactericidal effect was enhanced after adding sulbactam (4 mg/L). Bacterial growth were inhibited by 4 h in the antibiotic combinations (Zhu et al., 2022a).

Metabolomics was used to elucidate the pathways that resulted in synergistic effects of polymyxin-B and amikacin with or without sulbactam. Disequilibrium of key metabolic pathways was induced by polymyxin-B/amikacin/sulbactam; altered metabolites occurred with lipid, nucleotide, and amino acid metabolisms, as well as CCM and cell wall synthesis. The sequence of events is summarized in Table 3.

Lipopolysaccharide and peptidoglycan are the main components of cell wall. The resistance mechanism of *A. baumannii* to colistin is mainly due to the modification of lipoprotein A or the loss of LPS; polymyxin-B exerts its effect through electrostatic interaction with LPS. To counteract the effect of polymyxins, *A. baumannii* can increase its own cell survival by upregulating arginine biosynthesis to weaken the electrostatic interaction between colistin-LPS. Metabolomics and transcriptomics results showed that polymyxin-B monotherapy can reduce the synthesis rate of peptidoglycan and lipoprotein A (Han et al., 2019; Zhu et al., 2019; Zhao et al., 2021).

The effect of combination therapy on lipopolysaccharide and peptidoglycan metabolic pathways occurred earlier than that in monotherapy (Table 3). The combination of polymyxin-B/amikacin/sulbactam reduced the level of glucosamine-6-phosphate (GlcN6P), UDP-Glc and UDP-GlcA, which are important metabolites and precursors of glycosylated protein and lipid biosynthesis and related to cell wall maintenance (Zhao et al., 2014; Warth et al., 2015; Kuang et al., 2016). This may be due to the effect of sulbactam, since sulbactam can disintegrate the cell wall of *A. baumannii*, allowing companion antibiotics to enter the bacterial cell (Horii et al., 2002; Penwell et al., 2015). This shows that sulbactam in the combination therapy has the advantage of destabilizing cell wall synthesis.

The integrity and stability of the cell outer membrane play an important role in the normal growth of bacteria (Zhang and Rock, 2008). Polymyxin-B can disrupt the outer membrane of gram-negative bacteria, as well as phospholipid exchange, resulting in an increased cell permeability (Velkov et al., 2013; Zhao et al., 2021). However, due to the drug resistance in this isolate, polymyxin-B alone did not have much effect on the metabolites related to the outer membrane pathway. Interestingly, the combination of polymyxin-B/amikacin/sulbactam significantly affected the metabolisms of sn-glycero-3-phosphocholine, sn-glycero-3-phosphoethanolamine and N-methylethanolamine phosphate, which are three essential intermediates involved in glycerophospholipid pathway (Zhang and Rock, 2008; McHenry et al., 2012). This result demonstrated that polymyxin-B/amikacin/sulbactam combination can destabilize bacterial outer membrane to overcome the drug resistance caused by cell membrane modification (Loutet and Valvano, 2011).

As the cell wall and membrane stability of bacteria are destroyed, the permeability and integrity of the outer membrane are no longer stable, allowing for more drugs to enter the bacteria. Amikacin antibacterial mechanism is via the inhibition of protein synthesis that leads to cell death (Taber et al., 1987; Kotra et al., 2000). This mechanism of action is consistent with our result that a large number of amino acids and peptides were affected. Glutathione was significantly decreased by polymyxin-B/amikacin/sulbactam. It is an important marker of oxidative stress response. Previous studies have shown that polymyxins may induce oxidative stress through the formation of hydroxyl radicals and reactive oxygen species, which target DNA, RNA and lipids (Cabiscol et al., 2000; Sampson et al., 2012). The decrease in glutathione level is also an early activation signal of apoptosis; the subsequent generation of oxygen free radicals promotes cell death (Armstrong et al., 2002). Acetyl-CoA, an important intermediate in the synthesis of amino acids and fatty acids (Gest, 1987; Murima et al., 2014; Wolfe, 2015; Hussein et al., 2019b), was significantly disrupted by the triple-antibiotic combination. In addition, the triple-antibiotic combination perturbed some metabolites, which are all critical intermediates in the CCM pathway including tricarboxylic acid (TCA) cycle, pentose phosphate pathway and gluconeogenesis/glycolysis pathway, such as citric acid, oxalosuccinic acid, succinic acid and pyruvic acid (Table 3 and Figure 5).

Nucleotide metabolism pathway is essential for energy, lipid and protein biosynthesis (Moffatt and Ashihara, 2002; Armenta-Medina et al., 2014; Zhao et al., 2021). The triple-antibiotic combination disturbs the stability of pyrimidine and purine metabolism over a longer period of time. This will have a greater impact on the stability and normal growth of bacteria. In addition, nicotinamide as an ATP reserve pool (Hussein et al., 2019a), was also significantly affected by the combination of polymyxin-B/amikacin/sulbactam, suggesting that the energy balance in the bacteria was disoriented. The action of combination therapy was more sustained than that of single-agent administration. The addition of sulbactam provided stronger disruption than that of polymyxin-B/amikacin combination.

Our previous study demonstrated the synergistic effect of another triple-combination therapy consisting of polymyxin-B/meropenm/sulbactam (Menegucci et al., 2019; Fedrigo et al., 2021; Zhang et al., 2022; Zhu et al., 2022b) and demonstrated disruption of the metabolomic profile of a clinical *A. baumannii* isolate (Zhu et al., 2022c). The metabolites involved in the cell wall synthesis, outer membrane integrity and central carbon metabolism were primarily affected by polymyxin-B/meropenem or polymyxin-B/meropenem/sulbactam combinations. The addition of sulbactam to the double combination induced metabolic disruption at an earlier time. In contrast, the present study replaced meropenem with amikacin; this triple-antibiotic combination with amikacin also disrupted metabolisms of the nucleotides, amino acids and peptides. There is a stark difference in treatment effects for the two triple-antibiotic combinations that may explain why the combination with amikacin resulted in a better bactericidal effect and inhibition of mutant selection for their clinical regimens based on model-simulated parameters (Zhang et al., 2022; Zhu et al., 2022a).

In conclusion, the present study elucidated the effects of combination therapy on a clinical *A. baumannii* isolate. The metabolic pathway analysis showed that the effect of the combination is both enhanced and sustained. At the early stage, polymyxin-B disturbs the stability of cell membrane, making it easier for amikacin and sulbactam to enter the bacteria. Sulbactam further disrupts cell wall synthesis allowing amikacin to penetrate the cell and alter protein synthesis.

## Data availability statement

The original contributions presented in the study are included in the article/supplementary material, further inquiries can be directed to the corresponding authors.

## Author contributions

All authors listed have made a substantial, direct, and intellectual contribution to the work and approved it for publication.

## Conflict of interest

The authors declare that the research was conducted in the absence of any commercial or financial relationships that could be construed as a potential conflict of interest.

## Publisher’s note

All claims expressed in this article are solely those of the authors and do not necessarily represent those of their affiliated organizations, or those of the publisher, the editors and the reviewers. Any product that may be evaluated in this article, or claim that may be made by its manufacturer, is not guaranteed or endorsed by the publisher.

## Footnotes

^1^ https://www.metaboanalyst.ca/
